# Diagnostic and Therapeutic Challenges of Concurrent Intracranial Hemorrhage and Cerebral Venous Thrombosis in a Patient With Acute Lymphoblastic Leukemia: A Case Report and Literature Review

**DOI:** 10.7759/cureus.37482

**Published:** 2023-04-12

**Authors:** Beatrice E Torere, Joseph Weigold, Henry O Aiwuyo, Gabriel Alugba, Olanipekun Ntukidem, Jiahuai Tan

**Affiliations:** 1 Internal Medicine, North Mississippi Medical Center, Tupelo, USA; 2 Internal Medicine, Brookdale University Hospital Medical Center, Brooklyn, USA; 3 Internal Medicine, Delta State University, Abraka, NGA; 4 Clinical Research, St. Jude Children’s Research Hospital, Memphis, USA; 5 Hematology and Oncology, North Mississippi Medical Center, Tupelo, USA

**Keywords:** concurrent intracranial hemorrhage, cerebral venous sinus thrombosis (cvst), acute lymphoblastic leukemia (all), cerebral venous thrombosis, intracranial hemorrhage

## Abstract

Cerebral venous sinus thrombosis (CVST) is a cerebrovascular condition due to the thrombosis of cerebral venous sinuses, leading to intracranial hemorrhage, increased intracranial pressure, focal deficit, seizure, toxic edema, encephalopathy, and death. The diagnosis and therapeutic approach of CVST remain challenging because of its highly nonspecific clinical presentation including headaches, seizures, focal neurologic deficits, and altered mental status, etc. Anticoagulation is the mainstay of CVST treatment and should be started as soon as the diagnosis is confirmed. Here, we present the case of a 34-year-old male construction worker who presented to the emergency department with a complaint of right chest wall pain and swelling. He was admitted to the hospital following a diagnosis of anterior chest wall abscess and mediastinitis. During hospitalization, his complete blood count revealed pancytopenia with blast cells, and bone marrow biopsy revealed 78.5% lymphoid blasts by aspirate differential count and hypercellular marrow (100%) with decreased hematopoiesis. He developed concurrent CVST and intracranial hemorrhage while receiving CALGB10403 (vincristine, daunorubicin, pegaspargase, prednisone) with intrathecal cytarabine induction chemotherapy for acute lymphoblastic leukemia (ALL). The patient failed two standard chemotherapy for ALL and achieved remission while on third-line chemotherapy with an anti-CD19 monoclonal antibody, blinatumomab. Although this patient had an MRI scan of the brain with multiple follow-up non-contrast CT scans, it was CT angiography that revealed CVST. This showed the diagnostic challenge in CVST, with CT and MRI venography having excellent sensitivity in diagnosing CVST. Risk factors for CVST in our patient were ALL and its intensive induction chemotherapy with pegaspargase.

## Introduction

The diagnosis and therapeutic approach of cerebral venous sinus thrombosis (CVST) remain challenging because of its diverse clinical presentation. CVST is a cerebrovascular condition caused by thrombosis of the cerebral venous sinuses leading to intracranial hemorrhage, increased intracranial pressure, focal deficit, seizure, toxic edema, encephalopathy, and death [1].

CVST typically presents as a rare type of stroke, representing 0.5-1% of all strokes in adults and affecting all age groups [2]. It has a mortality of 30% and annual incidence ranging from 1.16 to 2.02 per 100,000. It is more common in females, with a median age of 34 years. Studies have shown that there is a 3:1 ratio of females to males in CVST [3,4]. This disproportion is likely due to hypercoagulability associated with pregnancy and puerperium and oral contraceptive use.

The most common sites for thrombus formation are the superior sagittal, transverse, and sigmoid sinus. The superior sagittal occlusion is associated with intracranial hemorrhage [5].

The pathogenesis of CVST is not fully understood due to the high variability of the venous system. However, two mechanisms are thought to contribute to the clinical presentation of cerebral venous thrombosis. First, thrombosis of cerebral veins blocks blood drainage from brain tissue, leading to increased venous and capillary pressure with disruption of the blood-brain barrier, causing vasogenic edema, with leakage of blood plasma into the interstitial space. The increased pressures in the venous system cause a continuous increase in intracranial pressure, localized cerebral edema, and intraparenchymal hemorrhage due to venous or capillary rupture. The second mechanism resulting in CVST is obstruction of the venous sinus leading to decreased cerebrospinal fluid absorption and increased intracranial pressure [6].

Multiple acquired and hereditary factors are associated with CVST and include surgery, pregnancy, postpartum state, oral contraceptives, obesity, head injury, bacterial infection, COVID-19 infection, COVID-19 vaccine, antithrombin III deficiency, protein C and protein S deficiency, factor V Leiden, prothrombin gene mutation, myeloproliferative diseases, celiac disease, malignancy, chemotherapy agents (L-asparaginase, all-trans retinoic acid, and cisplatin), and inflammatory diseases such as systemic lupus erythematosus, sarcoidosis, inflammatory bowel disease, granulomatosis with polyangiitis, and thromboangiitis obliterans [7-10]. The etiology of CVST is usually multifactorial, with up to 65% of patients with CVST having more than one risk factor [11]. The clinical signs are highly nonspecific, with headaches, seizures, focal neurologic deficits, and altered mental status representing the most common symptoms. The presence of parenchymal involvement, the interval from symptoms to diagnosis, and the site and extent of the thrombosis are important in the clinical presentation of thrombosis [1].

Due to its rarity and highly variable clinic presentation, a high suspicion index is required to diagnose CVST. It is life-threatening in the setting of delays in diagnosis or initiation of therapy. CVST has a diverse clinical course and subacute onset, which often leads to a delay in diagnosis with an average delay of seven days from the onset of symptoms [12]. Yet, prompt diagnosis and treatment are paramount to ensure survival and reduce complications.

## Case presentation

A 34-year-old male with a history of methamphetamine abuse, intravenous drug use, and asthma was admitted to the hospital with complaints of right chest wall pain and swelling of four days duration. He was a construction worker and thought his chest pain was due to muscle strain. The patient denied direct trauma to his chest, fever, chills, headache, dyspnea, cough, abdominal pain, nausea, vomiting, dysuria, and hematuria. Physical examination revealed normal vital signs and an awake, alert, and oriented male in no apparent distress. Neurological examination was benign, and cranial nerves were intact. There was swelling over the right upper anterior chest wall with associated warmth, erythema, and tenderness. He had multiple tattoos, with needle stick marks on the right forearm and anterior cubital fossa.

In the emergency department, blood specimen was obtained for laboratory Investigation, and he was started on intravenous (IV) piperacillin and tazobactam empirically. Initial laboratory evaluation was significant for raised white blood cell count, erythrocyte sedimentation rate, D-dimer, and ferritin. Furthermore, hemoglobin, blood iron, and total iron binding capacity were also decreased, and the urine drug screen was positive for amphetamines and methamphetamine (Table 1).

**Table 1:** Laboratory findings.

Test	Finding	Reference range
White blood cell count (×1,000/UL)	2.5	4.8–10.8
Hemoglobin (mg/dL)	6.1	14.0–18.0
Platelet count (×1,000/UL)	160	130–400
Erythrocyte sedimentation rate (mm/hour)	127	0–15
Creatinine kinase (IU/L)	184	50–170
D-dimer (µg/mL)	5.04	0.0–0.5
Prothrombin time (seconds)	12.6	9.0–12.0
International normalized ratio	1.16	0.8–1.1
Vitamin B12 (pg/mL)	272	180.0–914.0
Folate (ng/mL)	14.8	4.0–20.0
Blood iron (µg/dL)	33	50–212
Total iron binding capacity (µg/dL)	238	250–450
Iron saturation (%)	14	11–46
Transferrin	170	203–362
Ferritin (ng/mL)	2,090.0	23.9–336.2
Urine drug screen (ng/mL)	Amphetamine: 500; Methamphetamine: 500	<50
HIV1/HIV2	Negative	

**Figure 1:**
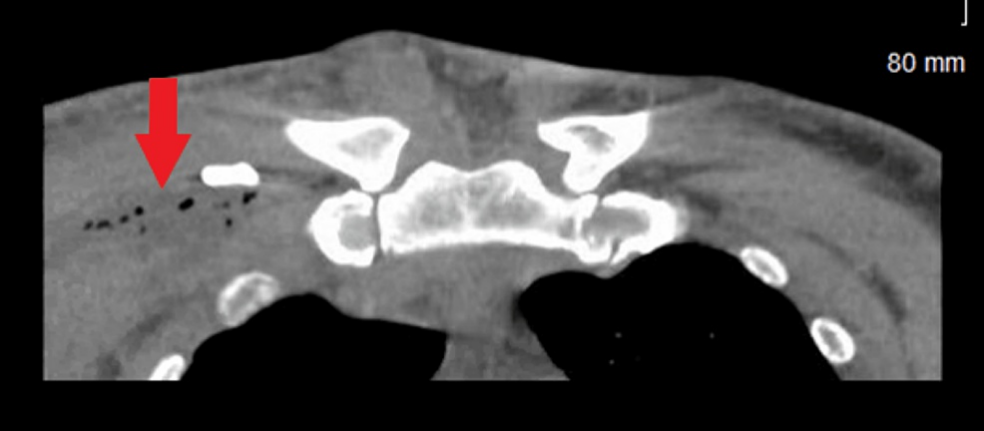
Computed tomography of the chest (coronal view). The red arrow indicates anterior chest wall with developing abscess and mediastinitis.

**Figure 2:**
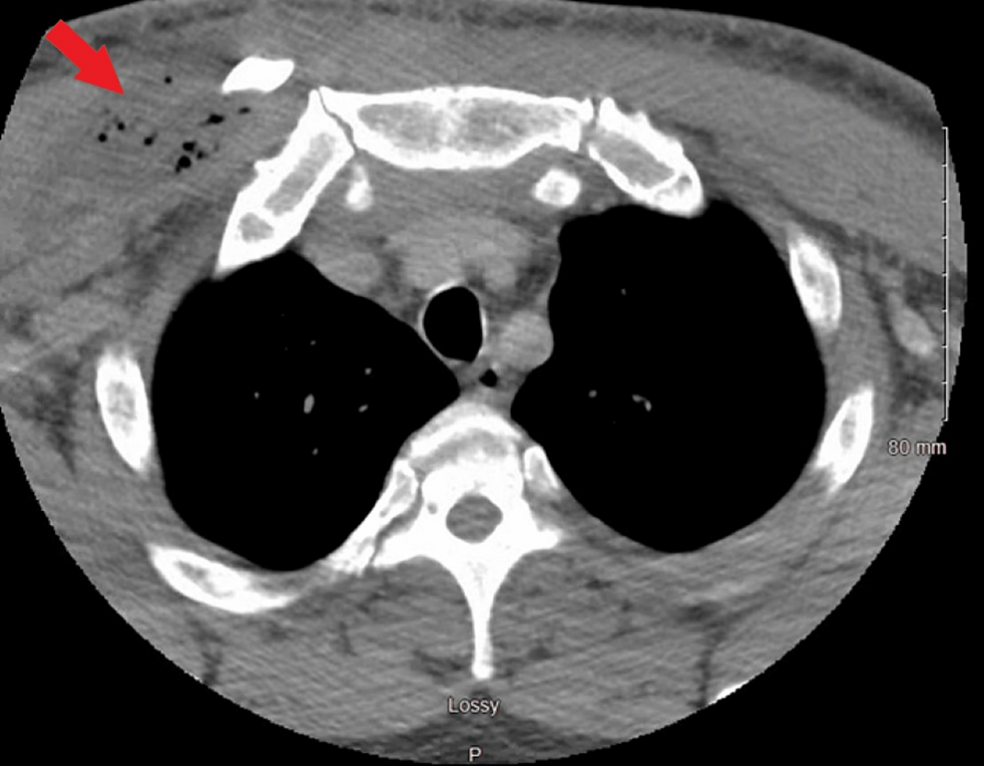
Computed tomography of the chest axial view reveals soft tissue swelling involving the right pectoralis muscle group extending into the anterior mediastinum, suggestive of a developing abscess with mediastinitis (red arrow).

Computed tomography (CT) of the chest revealed extensive soft tissue swelling involving the right pectoralis muscle group extending into the anterior mediastinum, suggestive of a developing abscess with mediastinitis (Figures 1, 2).

Empiric IV antibiotics were switched to IV cefazolin 2 g every eight hours. Cardiothoracic and infectious disease teams were consulted. The patient underwent an incision and drainage of the right chest wall abscess with drain placement. His abscess culture and blood culture grew methicillin-sensitive *Staphylococcus aureus* (MSSA). Echocardiography revealed normal heart chambers and valves with an ejection fraction of 55-60%, and no vegetation or abnormal intracardiac mass. The patient was managed with IV nafcillin 2 g every four hours for MSSA bacteremia. He was counseled on cessation of illicit drug use. His stool culture was positive for *Clostridioides difficile*, and he was started on oral vancomycin 125 mg every six hours for 10 days.

On day two of hospitalization, his complete blood count revealed pancytopenia and blasts. The pathologist ordered flow cytometry of peripheral blood smear, which revealed TdT+ lymphoid blasts comprising approximately 29% of analyzed white blood cells. A positive CD19 and CD34 but negative CD22, CD20, and CD38 were noted, along with hyperdiploidy. Fluorescence in situ hybridization results were negative for the rearrangement of *BCR-ABL1*, *ETV6-RUNX1*, and *KMT2A* (MLL). Bone marrow biopsy of his left and right iliac crest showed 78.5% lymphoid blasts by aspirate differential count, hypercellular marrow (100%) with decreased hematopoiesis, and normal marrow iron storage on the iron stain. The management of acute lymphoblastic leukemia (ALL) was discussed with the patient, and he was counseled on the chemotherapy adverse. The patient opted to proceed with treatment. Screening tests for HIV, hepatitis B and C, tuberculosis, and cerebrospinal fluid analysis were negative for infection.

**Figure 3:**
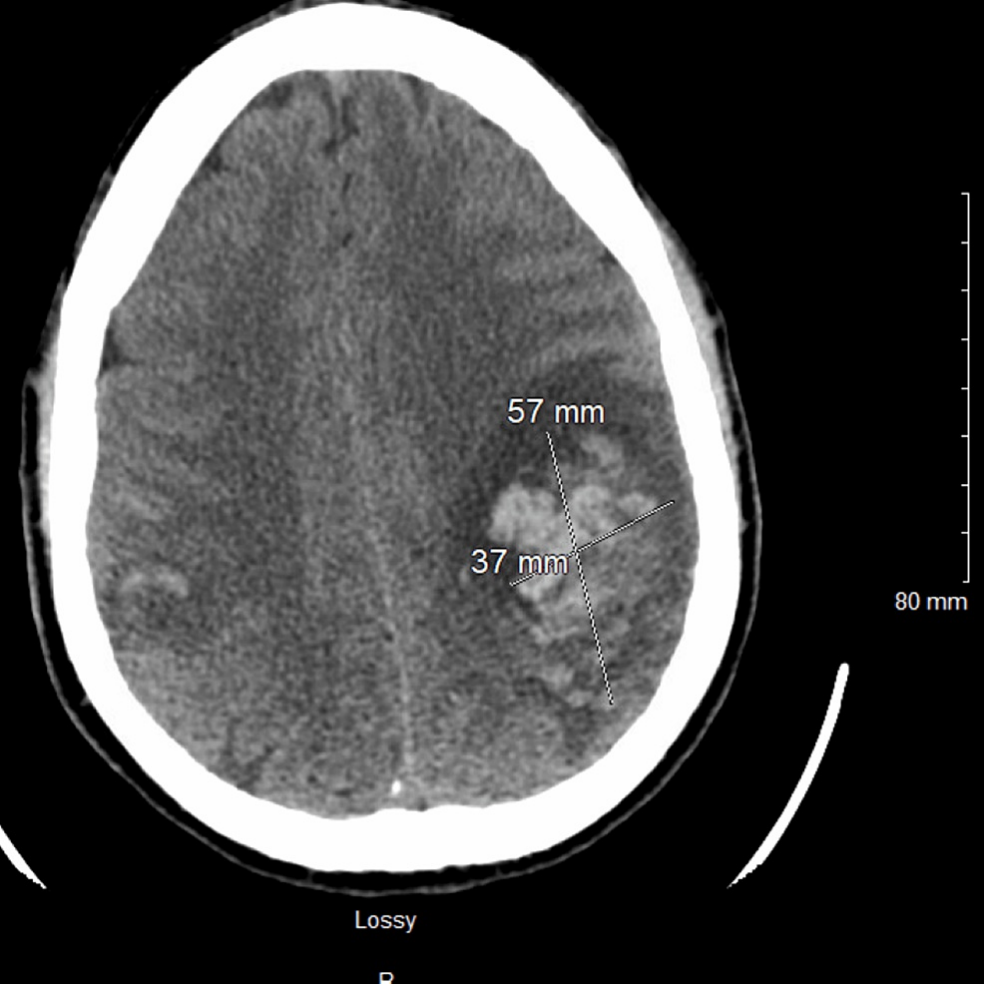
Computed tomography of the head (axial view). Left frontoparietal intraparenchymal hematoma measuring 37 mm × 57 mm.

A CALGB10403 (vincristine, daunorubicin, pegaspargase, prednisone) regimen and intrathecal cytarabine per protocol were started. On day eight of his chemotherapy treatment, he developed a headache. Magnetic resonance imaging (MRI) of the head was reported as normal; intrathecal methotrexate was administered. Repeat cerebrospinal fluid cytology showed no evidence of central nervous system leukemia or lymphoma. Two days later, he reported persistent and worsening headaches, with associated right arm numbness and dysarthria. Urgent CT of the head revealed a new acute intraparenchymal hematoma within the left cerebral hemisphere measuring 37 mm × 57 mm (Figure 3).

**Figure 4:**
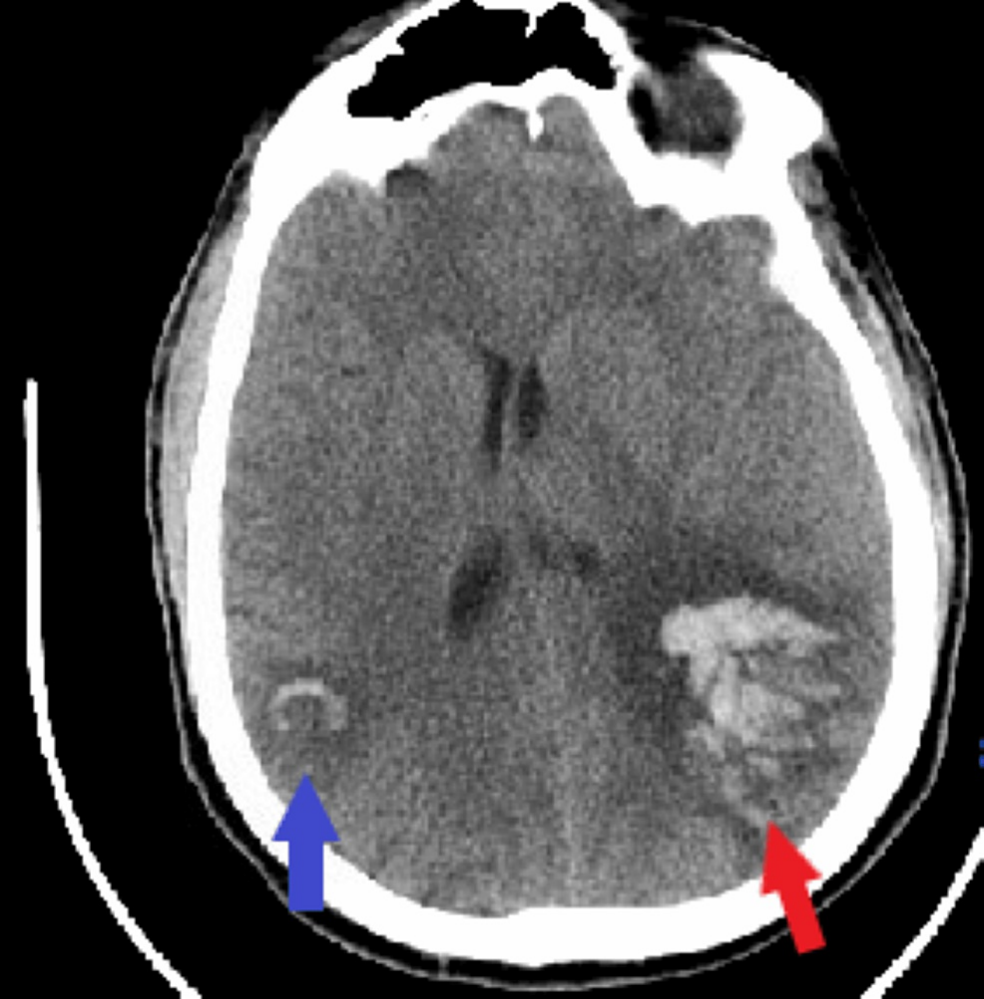
Computed tomography of the head (axial view). The blue arrow shows the right parietal lobe intraparenchymal hematoma, and the red arrow shows the left frontoparietal intraparenchymal hematoma.

Neurosurgery and neurology recommended avoidance of anticoagulants and antiplatelets due to intracranial hemorrhage. On day two in the intensive care unit (ICU), the patient developed a generalized tonic-clonic seizure. He received IV lorazepam followed by IV levetiracetam and IV lacosamide. Electroencephalogram (EEG) showed diffuse slowing of the EEG activity, right parietal, left frontal interictal epileptiform discharges, and intermittent high-amplitude delta activity, with triphasic morphology. Repeat CT head revealed a new right parietal lobe intraparenchymal hematoma, measuring approximately 2.6 × 2.2 cm, with surrounding vasogenic edema (Figure 4).

**Figure 5:**
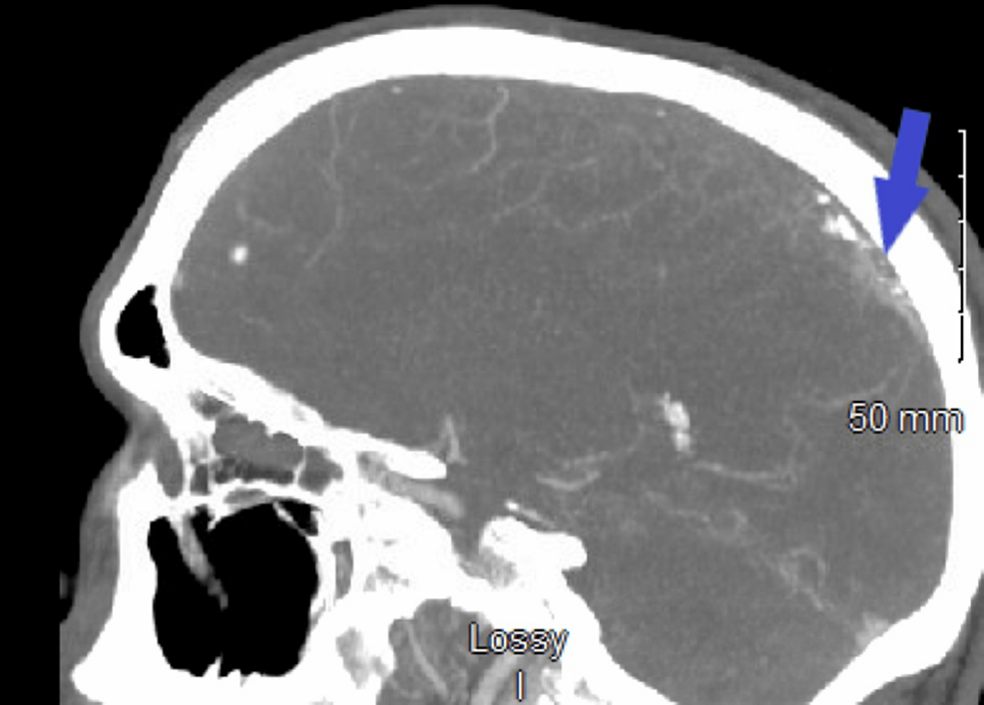
Computed tomography angiography of the head (sagittal view). The blue arrow shows superior sagittal sinus thrombosis.

**Figure 6:**
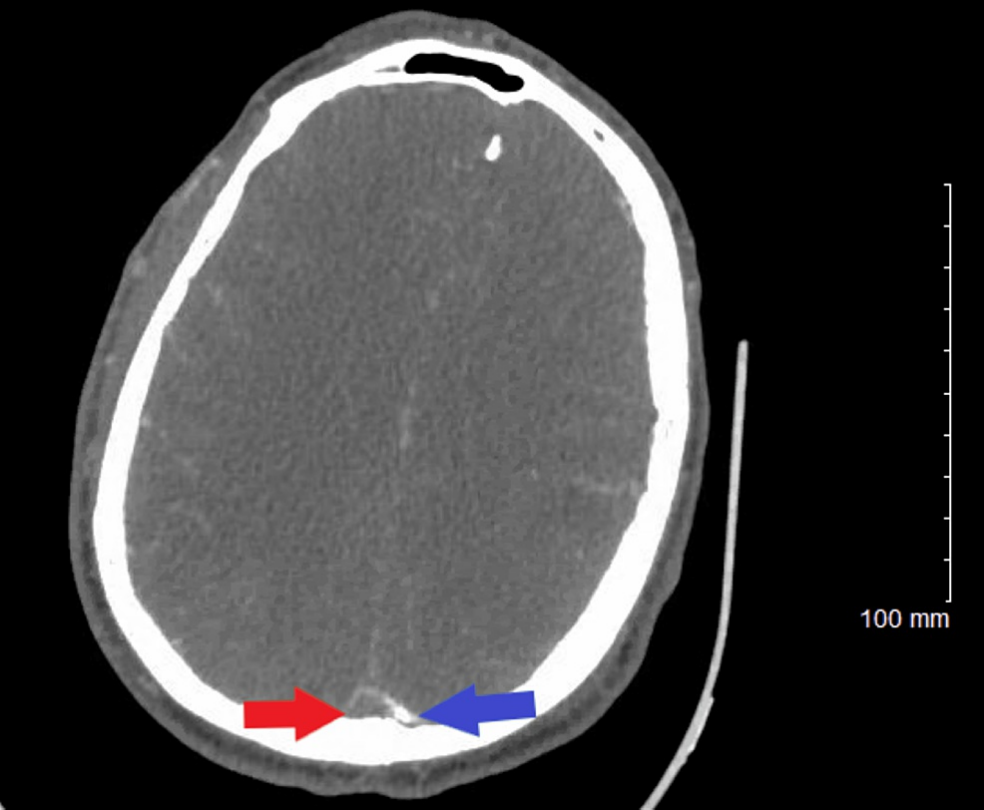
Computed tomography angiography of the head (axial view). The red arrow shows superior sagittal sinus thrombosis, and the blue arrow shows decreasing vessel patency.

On day three in the ICU, follow-up CT showed slightly increased vasogenic edema from the cerebral intra-axial hematomas; however, the midline shift remained unchanged, and the size of both hematomas was slightly decreased. About 16 hours later, CT angiography (CTA) of the head and neck revealed superior sagittal sinus thrombosis and bilateral cortical vein thrombosis, with left larger than right cerebral hemispheric hematomas probably reflecting hemorrhagic venous infarcts (Figures 5, 6).

Repeat echocardiography showed an ejection fraction of 50-60%, no cardiac atrial septal defect, no abnormal intracardiac mass, and normal pericardium. Palliative care was consulted for the goals of care discussion. The patient was started on IV heparin but developed worsening pancytopenia with a white blood cell count of 1,500/UL while on IV heparin. Heparin was switched to argatroban. Chemotherapy was suspended, and he received filgrastim. The enzyme-linked immunosorbent assay test was negative for heparin-induced thrombocytopenia antibody. Argatroban was changed to subcutaneous enoxaparin. He improved after three weeks in the ICU and was discharged to the hospital step-down unit. Unfortunately, a repeat bone marrow biopsy 34 days after starting chemotherapy revealed persistent ALL with 56% blasts. Due to severe complications from pegaspargase with cerebral vein thrombosis and suboptimal response to CALGB1040, his regimen was switched to HyperCVAD (hyperfractionated cyclophosphamide, vincristine, adriamycin, and dexamethasone). A follow-up bone marrow biopsy showed persistent refractory disease. HyperCVAD was changed to blinatumomab. Repeat CT of the head revealed resolution of the previous high attenuation within the bilateral parenchymal hematomas, decreasing vasogenic edema and mass effect, and no new anatomic abnormality in the interval.

Upon discharge, he was medically stable but with residual left-sided weakness and slurred speech. He was discharged home to continue blinatumomab outpatient. Repeat bone marrow biopsy showed complete remission with negative measurable residual disease. He was referred to an outside facility for stem cell transplantation.

## Discussion

It is important to have a high index of suspicion of CVST in patients with the aforementioned risk factors. The occurrence of simultaneous intracranial hemorrhage and CVST is rare, and the diagnosis and treatment of CVST in the setting of intracranial hemorrhage are challenging. Yet, rapid diagnosis and treatment are essential for survival. Literature review shows that the median delay from the onset of symptoms to diagnosis is seven days [13]. The risk factors in our patient included ALL and its intensive induction chemotherapy with L-asparaginase. Pegaspargase (polyethylene; PEG: *Escherichia coli*-derived L-asparaginase) is the first-line therapy for children and adults <65 years old with ALL. Pegaspargase is effective chemotherapy; however, despite the clinical benefit in ALL, it is associated with various adverse effects, including abnormal clotting studies and thrombosis [14]. On average, 3% of patients discontinued treatment due to intolerable adverse effects. During C10403 (vincristine, daunorubicin, pegaspargase, prednisone) induction chemotherapy. Our patient developed a hematoma with a large left frontoparietal central nervous system bleed/shift and edema. Shortly after, a right parietal bleed and seizure episodes led to the immediate discontinuation of pegaspargase therapy. A follow-up MRI of the head and four follow-up non-contrast CT scans of the head did not report CVST. CTA of the head and neck on day three of symptom onset revealed superior sagittal sinus thrombosis with associated hemorrhagic infarcts. He was treated with IV heparin.

The most common sites for thrombus formation are the superior sagittal, transverse, and sigmoid sinus. The superior sagittal occlusion is associated with intracranial hemorrhage [5].

When an occlusion occurs in the cerebral veins, the ensuing secondary venous hypertension is transmitted to the cortical veins, leading to the dilation and rupture of the fragile thin-walled cortical veins [15].

Due to its rarity and highly variable clinic presentation, a high suspicion index is required to diagnose CVST. It is life-threatening in the setting of delays in diagnosis or initiation of therapy. According to researchers, due to the diverse clinical course and often subacute onset, the average delay from symptoms to diagnosis of CVST is seven days [16,17]. Yet, prompt diagnosis and treatment are paramount to ensure survival and reduce complications.

Couturier et al. [18] reported their findings from the GRAALL study which revealed cerebral venous thrombosis occurring in 3.1% of adult patients during ALL induction therapy with prednisone, daunorubicin, vincristine, and L-asparaginase, as in the case of our patient who also had this induction therapy. In the CAPELAL trial [19], a 2.3% incidence of cerebral venous thrombosis was observed in patients who were on induction therapy with vincristine, idarubicin, L-asparaginase, and prednisone. Moreover, a 4.3% incidence was seen in 47 adult patients treated at the Dana Farber Cancer Institute [20]. Dubashi and Jain [21] reported a case of ALL in a 16-year-old male who was placed on induction therapy consisting of vincristine, L-asparaginase, daunorubicin, and steroids, and, subsequently, developed cortical venous thrombosis following induction therapy.

Neuroimaging and laboratory studies can help in the diagnosis of CVST [1]. In the past, D-dimer levels were useful as an initial screening test [17]. A 2004 study reported the sensitivity of D-dimer to be as high as 97.1% and specificity to be 99.1% [22]. However, recent studies have shown that up to 10% of patients with CVST have a normal D-dimer [17]. During the past decade, greater awareness of the disease has improved neuroimaging techniques, and more effective treatments have improved the outcome. In patients with CVST, 30-40% present with intracranial hemorrhage, which is often associated with superior sagittal occlusion [23].

A 2016 study showed that non-contrast CT (NCCT) as a first-line investigation has a high value in the diagnosis of CVST in the emergency setting [24]. The study reported that the sensitivity and specificity of NCCT for the overall presence of CVST were 85%, and 87%, respectively. However, four follow-ups of NCCTs did not report CVST in our patient.  NCCT can reveal findings of CVST, but in 10-40% of cases, NCCT shows normal findings [25,26].

Dimensional time-of-flight magnetic resonance venography (MRV) has excellent sensitivity and is the most currently used method for the diagnosis of CVST [1]. When using MRV, there is a direct sign of CVST demonstrated by high flow signal loss and fuzzy edges of a normally developed venous sinus or irregular lower blood flow signals [27,28]. Chronic thrombosed hypoplastic sinus presents with marked enhanced sinus and no flow on two-dimensional time-of-flight MRV, and contrast-enhanced MRI offers improved visualization of cerebral venous structures [1]. In patients with persistent and progressive symptoms despite medical treatment, repeated neuroimaging including CT venography (CTV) or MRV can help in identifying the development of a new ischemic lesion, intracranial hemorrhage, propagation of thrombus, edema, or other brain parenchymal lesions [29].

In our case, CTA of the head and neck was performed which revealed superior sagittal sinus thrombosis and bilateral cortical vein thrombosis, with left larger than right cerebral hemispheric hematomas. Cerebral angiography is helpful in the diagnosis of acute CVST, as CT or MRI may not provide adequate visualization of some cerebral veins, especially cortical veins and in some situations deep venous structures. Moreover, hypoplasia or atresia of cerebral veins or dural sinuses may lead to inconclusive results on MRV or CTV which can be clarified during the venous phase of cerebral angiography [1].

Findings from Haghighi et al. [30] revealed an overall mortality rate of 4.39%; however, some other studies showed the overall mortality rate to be between 9% and 15% [3,31].

Palazzo et al. [32] discovered a CVT recurrence rate of 2.6%, while Gosk-Bierska et al. [33] found a 2.2% annual sinus thrombosis recurrence rate. Similarly, Miranda et al. [34] noted a CVT recurrence rate of 2.2%. Peter et al. [35] observed a 1.8% CVT recurrence rate, whereas, in the VENOPORT study, 1.6% overall CVT recurrent thrombosis was reported [36]. In the ISCVT (International Study on Cerebral Vein and Dural Sinus Thrombosis) study, a large prospective multinational observational study, a 2.2% overall CVT recurrence rate was seen [3].

The mainstay of CVST treatment is anticoagulation, and concurrent CVST and intracranial hemorrhage is not a contraindication to treatment with an anticoagulant.

Management of CVST includes treating the underlying causes if known, control of seizure and intracranial pressure, and anticoagulant therapy. Endovascular thrombolysis or mechanical thrombectomy are treatment options for patients with CVST who develop worsening neurologic symptoms despite adequate anticoagulation therapy. Hemicraniectomy is indicated in patients with impending herniation.

## Conclusions

We highlight the importance of a multidisciplinary approach, including emergency physicians, internists, oncology, infectious disease specialist, neurologist, neurosurgery, and palliative care, to improve patient outcomes. There is a need to have a high suspicion of CVST in patients on asparaginase for treatment of ALL because prompt diagnosis and treatment with anticoagulants are paramount to prevent neurological morbidity and death. Concurrent CVST and intracranial hemorrhage is not a contraindication to treatment with an anticoagulant. Furthermore, we emphasize the need to evaluate the diagnostic accuracy of NCCT in diagnosing CVST.
